# Prevalence of probable depression and factors associated with mean Hopkins Symptom Checklist (HSCL) depression score among young women at high risk aged 15–24 years in Kampala, Uganda

**DOI:** 10.1371/journal.pone.0270544

**Published:** 2022-06-30

**Authors:** Onesmus Kamacooko, Daniel Bagiire, Francis Xavier Kasujja, Miriam Mirembe, Janet Seeley, Rachel King

**Affiliations:** 1 MRC/UVRI & LSHTM Uganda Research Unit, Entebbe, Uganda; 2 London School of Hygiene and Tropical Medicine, London, United Kingdom; 3 Institute for Global Health Sciences, University of California, San Francisco, San Francisco, CA, United States of America; University of the Witwatersrand, SOUTH AFRICA

## Abstract

**Background:**

In populations at high risk of HIV infection, rates of depression can be elevated with far-reaching effects on overall well-being. There is limited research on depression among young women engaged in high-risk sexual behaviour in low and middle-income settings. We investigated the prevalence, correlates and factors associated with mean HSCL depression score among young women at high risk (aged 15–24 years old) in Kampala, Uganda.

**Methods:**

We conducted a baseline analysis of a randomized controlled trial. Probable depression was measured using the 15-item Hopkins Symptoms Checklist for depression (HSCL). This checklist has been validated in Ugandan populations, and our reliability test gave a Cronbach alpha coefficient of 0.89. The test was administered to all the participants. Participants whose HSCL mean score was greater than 1.75 were categorized as having probable depression. Socio-demographics and behaviour data were collected and factors associated with mean HSCL depression score were analysed using multiple linear regression.

**Results:**

Data was available for 600 participants, mean age 20.4 (SD±2.44) years. The prevalence of probable depression was 56% (95% CI, 52%-60%). Probable depression symptoms were most prevalent among those who reported ever-experiencing violence from a sexual partner (64.7%), those aged between 20–24 years (58.2%) and those who reported more than 10 sexual exposures in the month prior to the interview (56.8%). At the adjusted analysis level, condom use during their last sexual intercourse prior to the survey decreased probable depression symptoms by 0.147 units compared to those who never used condoms (β = -0.147, 95% CI -0.266–0.027). Having experienced physical violence by a sexual partners increased mean HSCL depression score by 0.183 units compared to those who have never experienced violence (β = 0.183, 95% CI 0.068–0.300). Participants who reported ever using drugs of addiction had their mean HSCL depression scoreincrease by 0.20 units compared to those who have never used (β = 0.20,95% CI 0.083–0.317).

**Conclusions:**

Probable depression is high in this population and increased mean HSCL depression score is related to violence. Periodic screening for depression and interventions targeting depression, partner violence and risky sexual behaviours are recommended.

## Introduction

Depression is a growing health concern in both developed and developing countries [1–3]. Research has shown that young people are at the high risk of mental disorders and psychological distress [4–7]. There is general lack of awareness at the population level, limited training among health care providers and scarcity of resources including treatment opportunities that may hide the real burden of depression in developing countries [8–10]. There is therefore, a critical need for studies on mental health prevalence and associated factors [11].

The prevalence of depression is particularly high among adolescents [12–14], that can be related with a number of short term and long term outcomes such as poor academic performance, low self-esteem, joblessness, trauma, increased risk of suicide and impaired performance at workplaces [15, 16]. Depression can cause increased stress, dysfunction and exacerbate the affected person’s life situation [17]. A systematic review of global mental health among children and adolescents showed that the associated factors can be categorised into life-long factors such as a genetic background or exposure to harmful substances in utero and age-specific factors such as substance use, developmental-behavioural disorders among others [18]. According to studies that were carried out in Kenya, Uganda and South Africa, age-specific risk factors among adolescent girls and young women at high risk (YWHR) include poverty, lack of social capital, substance use and exposure to violence [19–21].

Mental health disorders, particularly among YWHR involved in sex work are generally under studied in sub-Saharan Africa (SSA). Although there is limited research on this topic, a study in Malawi found that YWHR who are school drop outs are more likely to suffer mental disorders compared to those attending schools [22] and it was further discovered that the risk factors for school drop outs are poverty, daily struggles and lack of social capital [22].

Depressive symptoms may compromise adaptive coping strategies and may result into limited decision-making capacity as documented among young Ugandan women, leading to risky sexual activities [23, 24]. HIV infection may predispose adolescents to depression and/or anxiety through stress-induced knowledge of HIV status, enacted or internalized stigma, blame, victimization and violence, as seen in Zimbabwe and South Africa [25, 26]. In keeping with the recent recommendations about setting programmatic agendas for mental health disorders in SSA, [27] understanding the burden and the corresponding protective and risk factors is very important [18]. We examine the prevalence of and associated factors for probable depression among young female sex workers aged 15–24 years in Kampala, Uganda. Findings from this study can inform policy makers and program managers to develop plans aimed at reducing the burden of mental health disorders among YWHR.

## Materials and methods

### Study design

This is a cross-sectional analysis of the baseline data of a randomised controlled trial titled “A Cognitive Behavioural and Structural HIV Prevention Intervention for Young Ugandan Women engaging in High Risk Sexual Behaviour (ZETRA-study for zero transmission)” ClinicalTrials.gov NCT03203200.

### Sample size and power

The sample of 644 participants provided 80% power with a type-I error rate of 5% (2-sided) to detect a 33% reduction in the number of unprotected sex episodes (UVI) in the intervention arm net of post-randomization reductions in the control arms. The sample size calculation accounts for a baseline frequency of 5 unprotected episodes per week; a total of 4 assessments including baseline; within-person correlation estimated at 0.3; a control-arm reduction in frequency of UVI of 15%; and over-dispersion of 3 with respect to a Poisson distribution, used as an approximation to the negative binomial model. This sample size computation was based on the primary outcome of the main study.

### Study setting

All study activities were conducted at the Good for Health Women Project (GHWP) clinic. The GHWP clinic was established in a peri-urban community in southern Kampala in 2008 under the Medical Research Council/Uganda Virus Research Institute and London School of Hygiene and Tropical Medicine (MRC/UVRI & LSHTM) Uganda Research Unit to study the epidemiology of HIV and sexually transmitted infections (STIs) and to implement HIV and STI prevention among high-risk women. Women attending the clinic engaged in risky sexual behaviours, such as sex with men for money, goods or favours; recruitment of women from commercial-sex hotspots has been described elsewhere [28]. All women were screened for eligibility and gave written informed consent before being enrolled in GHWP. Eligible women were then enrolled in the GHWP clinic irrespective of HIV status. The clinic offered routine HIV counselling and testing, syndromic management of STIs, family planning, antenatal care, free condoms, risk reduction counselling, counselling for excessive alcohol use, TB screening and treatment, ART and co-trimoxazole/dapsone preventive therapy. Enrolled women attended quarterly visits for HIV prevention and treatment services and study visits every six months.

### Study population

This study enrolled from the specialized clinic, GHWP, for high-risk women described above. The screening questions that identified women as eligible included: reported engaging in commercial sex (self-reporting being female sex workers or received money, goods, or other favours in exchange for sex in previous three months) or working in entertainment facilities, having had an STI in the last 3 months, number of times having sex with different partners per day, number of irregular and regular sexual partners.

Participants <18 years met criteria as being mature or emancipated minors as per guidelines of the Uganda National Council for Science and Technology (UNCST) [29]. According to the UNCST guidelines, “*emancipated minors are individuals below the age of majority who are pregnant*, *married*, *have a child or cater for their own livelihood*.” As the age of majority in Uganda is 18 years, study participants aged 15–17 years fell into this category as they cater for their livelihood and were in some cases pregnant. The UNCST guidelines allow such participants to “independently provide informed consent to participate in research if: a) in the view of the REC [Research Ethics Committee], the research is not objectionable to parents or guardians (established by the REC with evidence from the community); and b) the research protocols include clear justification for targeting mature and emancipated minors as participants; and a clear justification for not involving parents or guardians in the consent process.

### Community Advisory Board (CAB)

Because of the vulnerable nature of some of our participants, we formed both a CAB with 13 members and a youth advisory board (YAB). The CAB was composed of gender and age balanced members of the district political and technical leadership i.e. the division [LCIII], Councilor, Local Council II and the Local Council Chairperson, a representative from Nsambya Hospital, a representative of the police, a representative from the Non-Governmental Organization Women at Work International (WAWI) an organization where sex workers work, two peer educators and GHWP staff. The CAB was particularly sensitive to the illegal nature of the study participants activities and any potential increased risks to the participants were reported to the study manager for action. Those that needed medical attention would be referred to Nsambya Hospital for further management. The CAB and YAB provided ongoing guidance and support on the implementation of the study and other ongoing research activities taking place at the GHWP. The YAB paid particular attention to issues regarding younger aged participants. Both boards would review all study procedures and provide feedback on the content and cultural appropriateness of the study, assessment measures and any Human Subjects issues.

### Measurement of the study outcome variable

The outcome of interest was probable depression, which was measured using the modified version of the Johns Hopkins Symptoms checklist for depression (JHSCL). This 15-item scale checklist quantifies depression symptoms and has previously been validated among East African populations [30]. The dependent variable used in this study was the mean HSCL depression score on the JHSCL-15, calculated for each respondent by summing item responses and dividing by 15. Our test for reliability for the JHSCL gave a Cronbach alpha coefficient of 0.89. The tool was administered to all the participants at baseline, and all those participants whose mean JHSCL score with a cut off of greater than 1.75 were categorized as having probable depression [31].

### Independent variables

Baseline characteristics of the participants were considered for this analysis. The sociodemographic characteristics considered were: participant’s age, level of education, religion, and tribe; and the behavioural characteristics were number of sexual partners ever had sex with, condom use in the last sexual intercourse, drug use, physical violence from the sexual partner and the number of episodes of sexual intercourse in the previous month.

### Data management and statistical analysis

Data was collected and entered by research staff on Case Report Forms and/or Android electronic tablets (hand-held computers) using Audio Computer Assisted Self Interviews (ACASI), a computer-assisted personal interviewing software. The mental health data and social demographic data were merged into one database, cleaned, and exported to STATA 15.0 (StataCorp, College Station, TX, USA) for further management and analysis. The participants’ characteristics were summarized in descriptive terms such as mean, median, standard deviations (SD) or percentage, as appropriate. Linear regression analysis was used to determine factors associated with mean HSCL depression score. Assumptions for linear regression assessed: linearity was checked by a scatter plot of the residuals against the fitted values, and it depicted no significant outlier; normality of distribution checked by a histogram, and well fitted the normal distribution curve. Furthermore, we checked for heterogeneity and there was no evidence of heterogeneity in the data. Bivariate/simple linear regression analysis was conducted and variables with p-value < 0.15 were considered for multiple linear regression. Multiple linear regression analyses were used to identify factors independently associated with mean HSCL depression score. Variables that had a P-value <0.05 were considered significantly associated with mean HSCL depression score. We further presented a dot plot for mean HSCL depression score by age group.

### Ethics approval and consent to participate

The study was conducted in compliance with country-specific laws and regulatory requirements. The conduct of the study also adhered to the GCP principles laid out by the International Conference on Harmonization (ICH) and the Declaration of Helsinki. The local IRB in Uganda at Uganda Virus Research Institute (GC/127/16/08/527) and the Uganda National Council for Science and Technology (HS1886) approved the study protocol and informed consent forms prior to enrolment. We obtained informed consent for all participants and as per the guidelines of Uganda National Council of Science and Technology (UNCST) including the “emancipated minors”.

## Results

**Fig 1:**
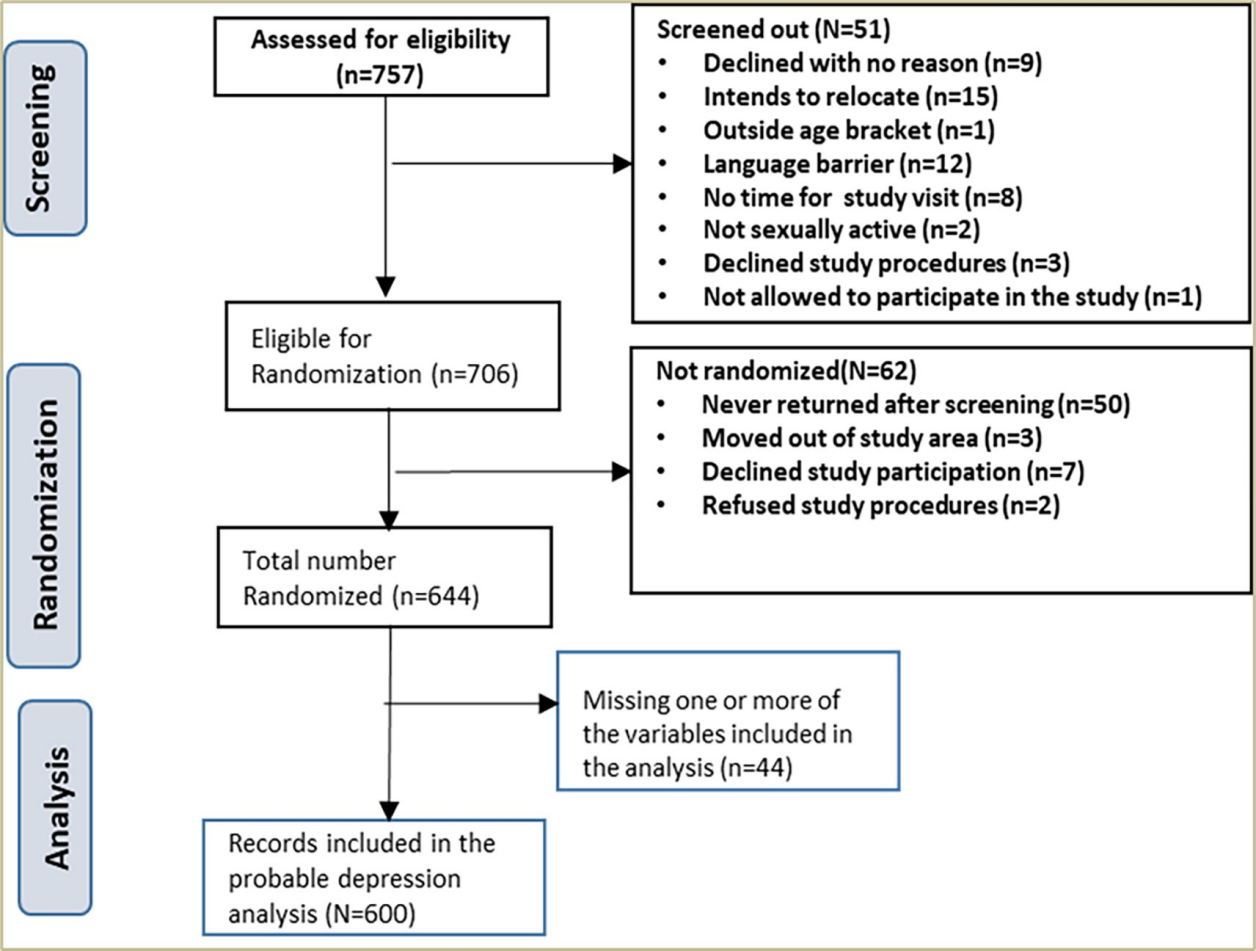
Screening profile of study participants.

Between the period November 2016 to February 2019, a total of 757 were cumulatively assessed for eligibility of whom 706 were eligible for randomization. A total of 51 participants were screened out. Out of the 706, a total of 644 were randomized of whom 600 (93.2%) were included in the probable depression analysis. Details Fig 1.

### Baseline characteristics

A total of 600 participants were included in the statistical analysis. The mean (±SD) age of study participants was 20.4 (± 2.44) years, with slightly more than a half [325 (54%)] aged between 20–24 years. Three hundred and thirty-three (56%) reported primary level as the highest level of education, 317 (53%) reported to have ever experienced physical violence from their sexual partner, 261(44%) reported to have had sex with 5–49 male sex partners. Three hundred eighty-two (64%) participants reported condom use during their last sex intercourse encounter before interviews. Please see details in Table 1.

**Table 1:** Descriptive and multivariable regression model of the factors associated with mean HSCL depression symptoms score among young women at high risk aged 15–24 years in Kampala, Uganda.

Variable	Overall N (col %)	Probable depression n (row %)	Simple LR β (95% CI)	P-value	Multiple LR β (95% CI)	P-value
All participants	600	335(55.8)				
**Age of the participant**						
15–19	275(45.8)	146(53.1)	Ref			
20–24	325(54.2)	189(58.2)	0.088(-0.026–0.202)	0.131	0.050(-0.062–0.162)	0.382
**Level of education**						
Primary Education	333(55.5)	201(60.4)				
Completed O-level or Beyond	267(44.5)	109(50.2)	-0.134(-0.248 - -0.020)	0.021	-0.103(-0.214–0.009)	0.072
**Religion**						
Catholic	227(37.8)	120(52.9)				
Protestant	106(17.7)	60(56.6)	0.020(-0.142–0.186)	0.795		
Muslim	173(28.8)	101(58.4)	0.060(-0.081–0.200)	0.403		
Born Again	80(13.3)	44(55.0)	0.027(-0.154–0.208)	0.769		
Other	14(2.3)	10(71.4)	0.307(-0.076–0.691)	0.116		
**Tribe**						
Muganda	303(50.5)	163(53.8)				
Munyankole	74(12.3)	49(66.2)	0.204(0.0024–0.384)	0.026	0.160(-0.017–0.336)	0.077
Musoga	41(6.8)	20(48.8)	-0.018(-0.250–0.212)	0.875	-0.022(-0.247–0.204)	0.851
Other Ugandan	152(25.3)	84(55.3)	0.132(-0.006–0.269)	0.769	0.114(-0.019–0.248)	0.094
Non Ugandan	30(5)	19(63.3)	0.234(-0.031–0.500)	0.116	0.231(-0.026–0.489)	0.078
**Number of male sex partners ever had sex with**						
1–4 Partners	133(22.2)	65(48.9)				
5–49 partners	261(43.5)	153(58.6)	0.142(-0.006–0.291)	0.059	0.107(-0.038–0.252)	0.149
50 +Partners	206(34.3)	117(56.8)	0.120(-0.035–0.274)	0.128	0.070(-0.084–0.223)	0.373
**Condom use during the last sexual intercourse**§						
No	196(32.7)	128(65.3)				
Yes	382(63.7)	192(50.3)	-0.207(-0.328 - -0.085)	0.001	**-0.147(-0.266 - -0.027)**	**0.016**
Dont remember	22(3.7)	15(68.2)	-0.049(-0.360–0.261)	0.756	0.018(-0.286–0.321)	0.909
**Ever used drugs of addiction**§						
No	339(56.5)	170(50.2)				
Yes	261(43.5)	165(63.2)	0.281(0.169–0.394)	<0.001	**0.200(0.083–0.317)**	**0.001**
**Ever experienced physical violence from the sexual partner**§						
No	283(47.2)	130(45.9)				
Yes	317(52.8)	205(64.7)	0.269(0.157–0.381)	<0.001	**0.183(0.068–0.300)**	**0.002**
**Number of sex intercourse in the last month**						
0–1 times	82(13.7)	41(50)				
2–9 times	247(41.2)	140(56.7)	0.104(-0.073–0.281)	0.249		
10+ times	271(45.2)	154(56.8)	0.120(-0.05–0.300)	0.180		

LR: Linear Regression; β Beta coefficients; CI: Confidence Interval; § significant at P < 0.05; N = 600

### Prevalence of probable depression and factors associated with mean

#### HSCL depression score

**Fig 2:**
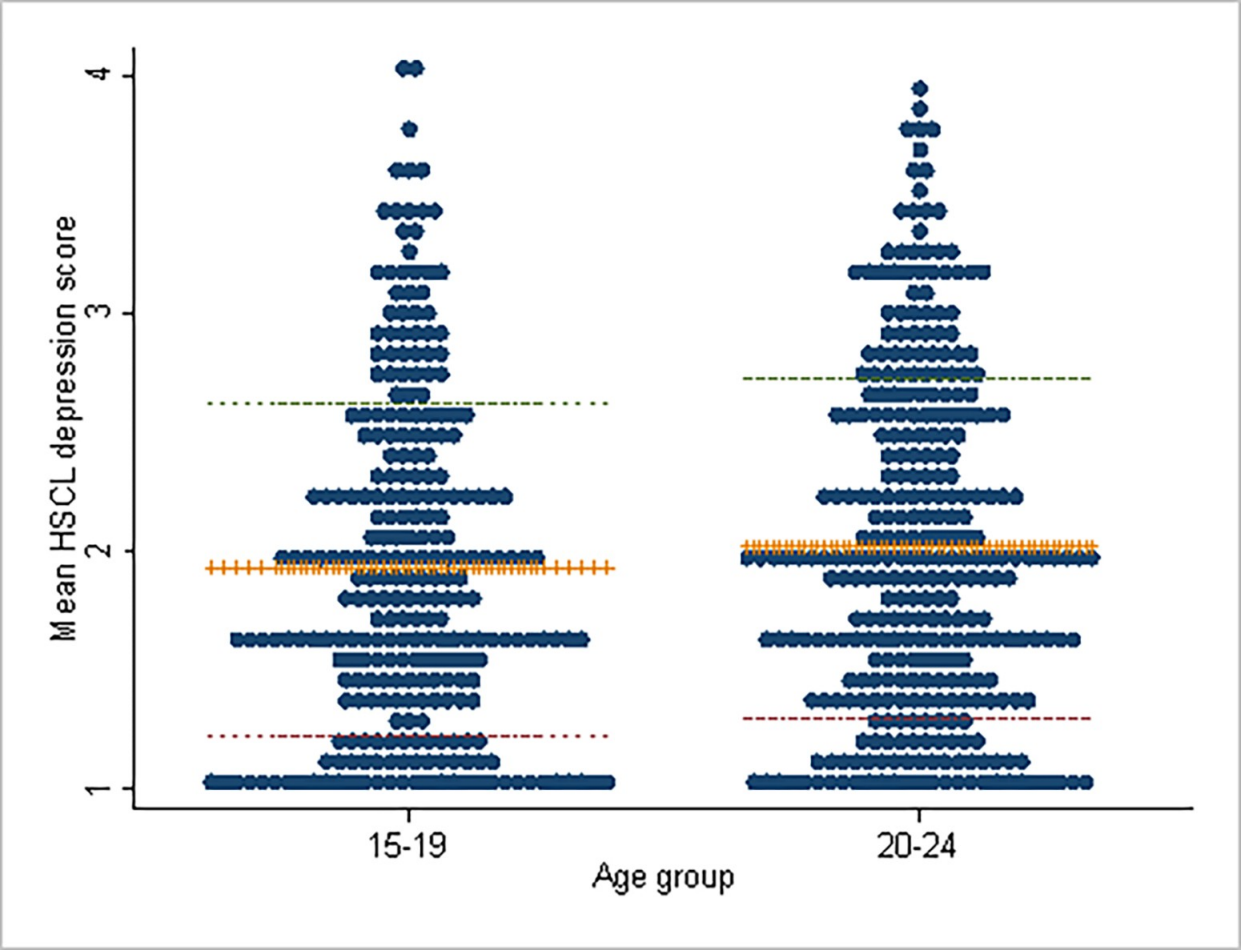
Distribution of mean HSCL depression score by age group. +++++ Represents average HSCL depression score.

Of the 600 participants that were included in the analysis, 335 (56%,95% CI:52%-60%) were categorized as having probable depression. The average JHSCL mean score was 1.98 with a standard deviation of 0.71. The mean score was higher among those aged 20–24 years compared with those aged 15–19 years (Fig 2), but this finding was not statistically significant (2.02 V^s^ 1.93, t-statistic -1.51; P = 0.131). At the bivariate linear regression level, we found that the level of education; age; condom use during the last sexual intercourse prior to the interview, ever used drugs of addiction and ever experience physical violence from a sexual partner met the criteria for inclusion in the multiple linear regression model.

At the adjusted analysis level, condom use during their last sexual intercourse prior to the survey decreased mean HSCL depression score by 0.147 units compared to those who never used condoms (β = -0.147, 95% CI -0.266–0.027). Having experienced physical violence from a sexual partner increased mean HSCL depression score by 0.183 units compared to those who have never experienced violence from sexual partners (β = 0.183, 95% CI 0.068–0.300). Participants who reported ever using drugs of addiction had their mean HSCL depression score increase by 0.20 units compared to those who had never used (β = 0.20,95% CI 0.083–0.317). Details Table 1.

## Discussion

We report on the prevalence probable depression and factors associated with mean HSCL depression score among young women at high risk in Kampala, Uganda. The findings of our study reveal that the prevalence of probable depression (56%) is higher than the results of a similar study (36%) carried out among the out of school adolescents and young women in Tanzania [32]. This variation could be explained by the differences in the populations studied. Whereas our study was conducted among high-risk adolescents, the study in Tanzania was conducted among a general population of same age who were not attending school. In studies carried out among high risk populations in Malawi, lower prevalence’s were reported at 47% and 49% compared to our study [33, 34]. In other studies conducted among female sex workers in Nepal and Mexico, very high rates of probable depression were found, ranging between 80% and 90% [35, 36]. Some of the variations in the prevalence rates might be explained by the differences in the populations studied especially with those in the general populations, as our study was carried out among young women at high-risk. For the studies carried out in similar populations, the differences might be explained by the measurement scales. Whereas we used the John Hopkins Symptoms checklist for depression (JHSCL), some studies used the Centre for epidemiological studies depression (CESD) scale.

We found that participants who experienced physical violence from sexual partners had increased mean HSCL depression score by 0.183 units compared to those who have never experienced violence from the sexual partners. Our findings are consistent with the results of several studies carried out in eastern and southern Africa where violence from sexual partners has been linked to mental health challenges [32, 37–39]. This could be attributed to the fact that some women depend on their partners for survival and this may lead to partner violence [40–42] which later leads to depressive symptoms. Putting in place policies that ensure women’s empowerment, gender equality and reduction of partner violence protection among the YWHR would have the potential to promote mental health [43]. Health practitioners should be aware that violence among adolescents might result in negative consequences in adulthood.

Participants who reported using condoms during their last sexual intercourse were less likely to suffer depressive symptoms compared to those who never used condoms during their last sexual intercourse. In other studies carried out in Uganda and Sub Saharan Africa reported that not using a condom was strongly related to probable depression [23, 44–48]. This could be attributed to the fact that YWHR have less power to negotiate less risky sexual behaviours [49] and the feelings of guilt, hopelessness, helplessness, and passive thoughts of contracting disease and death might explain the increased risk of probable depression.

The results further showed that participants who reported ever using drugs of addiction had their mean HSCL depression score increase by 0.20 units compared to those who have never used. These finding are consistent with the findings that reported that there was a significant relationship between substance use and probable depression symptoms [50, 51].

### Study limitations

We consider the way we defined probable depression as the main limitation of this study. This was based on the screening of the participants as opposed to the formal psychiatric evaluation. Research has shown that screening instruments tend to over-estimate the prevalence probable depression by a factor of two to three [30]. It should also be noted that there is no consensus regarding which of depression scales are optimal to measure depression in African settings. We used the HSCL, which has been validated in similar populations in East Africa [52].

## Conclusions

The high prevalence of probable depression in this study calls for urgent preventive and treatment interventions for optimal mental health aimed at YWHR in Uganda. Continued advocacy and increasing the use of the new national mental health act signed into force on 18^th^ February 2021 as well as focusing on healthy living and increasing efforts aimed at YWHR communities in Uganda especially on the effects of drug use of addiction and high risk sexual activities [53].
